# Effects of the Prior Use of Statins on Head and Neck Cancer Risk: A Hospital-Based Case–Control Study

**DOI:** 10.3390/ph15050579

**Published:** 2022-05-06

**Authors:** Constanza Saka-Herrán, Enric Jané-Salas, Antonio Mano-Azul, Aina Torrejón-Moya, Albert Estrugo-Devesa, José López-López

**Affiliations:** 1 Department of Odontostomatology, Faculty of Medicine and Health Sciences (Dentistry), University of Barcelona, 08970 Barcelona, Spain; constanzasakah@gmail.com (C.S.-H.); enricjanesalas@ub.edu (E.J.-S.); aina.torrejon@gmail.com (A.T.-M.); albertestrugo@ub.edu (A.E.-D.); 2 Oral Health and Masticatory System Group (Bellvitge Biomedical Research Institute) IDIBELL, Faculty of Medicine and Health Sciences (Dentistry), University of Barcelona, 08970 Barcelona, Spain; 3 Department of Oral Surgery, Oral Medicine, and Maxillofacial Surgery, Egas Moniz Higher Education School, Campus Universitario, Quinta da Granja, 2829-511 Caparica, Portugal; antonio.azul@zonmail.pt; 4 Faculty Director & Head of Service of the Medical-Surgical Area of Dentistry Hospital, University of Barcelona, 08970 Barcelona, Spain

**Keywords:** statins, hydroxymethilglutaryl-CoA reductase inhibitors, head and neck cancer, head and neck neoplasms, cancer, risk

## Abstract

Mechanisms related to the potential beneficial effects of statins on cancer are mainly related to the inhibition of the mevalonate pathway. The purpose of this study was to assess the association between prior use of statins and the risk of head and neck cancer. A hospital-based case–control study was conducted at the Dentistry Hospital of the University of Barcelona, including 101 incident cases of head and neck cancer and 101 controls matched to cases by age and sex. Multivariate logistic regression models were used to assess the association between prior statin exposure and head and neck cancer risk. Of the 202 patients included in total, 28.2% had previously received prescriptions for statins. Prior use of statins was found in 25.7% of cases and 30.7% of controls. Exposure to statins was not associated with head and neck cancer risk (OR = 0.72; 95% CI 0.28–1.84; *p* = 0.49). There was also no time- or dose-dependent association. Similar trends were observed when analyzed by subsites of cancer and recurrence rate. Our findings do not support a beneficial effect of prior statin exposure on head and neck cancer risk. Future research relying on observational data should emulate randomized clinical trials before clinical implications for repurposing drugs can be drawn.

## 1. Introduction

Statins are a group of lipid-lowering drugs that inhibit cholesterol biosynthesis by inhibiting 3-hydroxy-3-methyglutaryl-coenzyme A (HMG-CoA) reductase, which leads to the inhibition of L-mevalonate synthesis and subsequently decreases the hepatocellular cholesterol production [1]. Several studies have shown that statins have pleiotropic effects that are not limited to lowering cholesterol levels, with a wide range of anti-inflammatory, anti-tumor, anti-thrombotic and immunomodulatory effects [1].

Mechanisms of action underlying the potential antitumor effects of statins on cancer are mainly related to the inhibition of the mevalonate pathway [2]. Mevalonate is synthesized from HMG-CoA by HMG-CoA reductase and further metabolized to farnesyl pyrophosphate (FPP), a precursor of cholesterol, which is also converted to geranyl-geranyl-pyrophosphate [3,4]. These isoprenoids are used for the prenylation of proteins involved in several intracellular mechanisms [4]. Among them, Ras and Rho proteins seem to be the target proteins in the anti-tumor activity of statins. By inhibiting their prenylation, several key intracellular pathways are affected, including membrane integrity, cell signaling, protein synthesis and cell cycle progression [3,5].

Ras proteins are critical components of signaling pathways which control cell proliferation, differentiation and survival by activating the PI3K and Akt/PKB pathways [5]. The activating mutations of Ras genes are found in approximately 30% of all human cancers, and their prenylation seems to be an important step in the transformation of cells [5]. Rho proteins are implicated in the regulation of different cellular processes including cell adhesion, cell motility and cell proliferation [5]. An overexpression of Rho proteins has been observed in a variety of cancers and associated with increased tumor invasion [5]. The inhibition of Rho protein prenylation results in the inhibition of invasiveness and metastatic properties of tumor cells [5].

Several studies have established that the mevalonate pathway is increased in different types of cancer, including: leukemia, lymphoma, breast, liver, pancreatic, esophageal and prostate cancer [4]. Statins, as inhibitors of the mevalonate pathway, might be considered as chemopreventive agents or as adjuvants in cancer treatment by their interaction with essential cellular functions such as cell proliferation and differentiation [4]. In this line, data provided by observational studies have shown that statin use is associated with a lower risk of esophageal cancer (OR = 0.82; 95% CI 0.70–0.88) [6], improved cancer-specific survival from kidney cancer (HR = 0.67; 95% CI 0.47–0.94) [7] and improved survival in patients with colorectal cancer (HR = 0.8; 95% CI 0.79–0.86) [8]. Among women with breast cancer, statin use has been associated with improved overall survival (HR = 0.66 95%; CI 0.44–0.99) and improved recurrence-free survival (HR = 0.64; 95% CI 0.53–0.79); however, the benefit for recurrence-free survival was observed only for lipophilic statin exposure (HR = 0.72; 95% CI 0.59–0.89), not for hydrophilic statin use (HR = 0.80; 95% CI 0.44–1.46) [9].

Regarding head and neck cancer, results from the systematic review by Pavan et al. [10] show that statins decrease cell proliferation, cell differentiation, cell growth, cell progression and metastasis of head and neck squamous cell carcinoma (HNSCC) cell lines. Most of the studies included have shown that statins used alone were cytotoxic to HNSCC and reduced cell viability to less than 50% in a dose-dependent manner [11,12,13]. Additionally, some studies have demonstrated cell accumulation in the G0/G1 phase [12,14,15], showing that statins could control the cell cycle and apoptotic signaling, which has led to the conclusion that statins may have a potential role as adjuncts to standard therapies for HNSCC [10].

Although in vitro/in vivo studies have shown the anti-tumor effects of statins on head and neck cancer, the evidence provided by clinical trials and observational studies is limited [16]. The available evidence goes back no further than 2017 and, to date, only one study has assessed the association between statin use and head and neck cancer risk [17]. The study included 5515 patients with head and neck cancer and 5515 controls matched to cases by propensity score. Results showed that prior statin exposure was associated with a lower risk of head and neck cancer (OR = 0.86; 95% CI 0.77–0.95). Specifically, the observed associations were significant for regular use of statins and higher doses [17].

Given the increasing amount of evidence regarding drug repurposing in the prevention and treatment of cancer, driven by the improved understanding of the hallmarks of cancer and cancer-specific biological pathways [18], we have designed a case–control study to assess the potential beneficial effects of certain drugs, including statins, on head and neck cancer risk, under the hypothesis that prior statins use will be associated with a lower risk of head and neck cancer. The main objective of the study was to assess the association between prior statin exposure and head and neck cancer risk.

## 2. Results

### 2.1. Characteristics of Cases and Controls and Factors Related to Head and Neck Cancer

The sample included 101 patients with head and neck cancer (cases) and 101 controls matched to cases by age and sex (*n* = 202). Compared to controls, cases had a lower educational level and a lower monthly income (Table 1). Regarding the behavioral habits of cases and controls, 71.3% of cases had a history of tobacco smoking, with 58.4% being former smokers, while almost 50% of controls had no previous history of tobacco use. Furthermore, 69% of cases reported a smoking history of more than 20 cigarettes per day, which was significantly higher than in controls (24.1%) (*p* = 0.02) (Table 1). Cases were also more likely to have higher levels of alcohol intake than controls (23.8% and 4%, respectively) (*p* = 0.01). On the other hand, the percentage of patients with a healthy diet was significantly higher in controls (38.6%) than in patients with head and neck cancer (12.7%), who mainly maintained a poor diet (46.8%) (Table 1). Similarly, controls reported a higher regular physical activity of two or more times per week than cases (73.2% and 52.3%, respectively). The prevalence of cardiovascular disease was higher in cases than in controls, while controls had a higher prevalence of type 2 diabetes mellitus (DM-2) (Table 1). After adjusting the multivariate logistic regression model, factors significantly associated with higher odds of head and neck cancer were low (OR = 39.4; 95% CI 4.08–381.3) and middle income (OR = 8.86; 95% CI 1.01–78.0), being an ex-smoker (OR = 2.36; 95% CI 1.01–5.57), smoking >20 cigarettes/day (OR = 8.66; 95% CI 1.38–54.2) and excessive alcohol consumption (OR = 10.1; 95% CI 2.10–49.3). Compared to a poor diet, having a healthy diet was significantly associated with lower odds of head and neck cancer (OR = 0.29; 95% CI 0.10–0.84) (Table 1).

### 2.2. Cancer History of Patients Diagnosed with Head and Neck Cancer

Cases (*n* = 101) were patients diagnosed with primary head and neck cancer between 2014–2021; 68.3% were men; and the mean age was 66 years (Table 1). Most patients had cancer of the oral cavity (49.5%), followed by cancer of the larynx (26.7%) (Table 2). According to the TNM classification, 62.4% of head and neck cancer patients were diagnosed at an advanced stage of disease (III or IV). When comparing the stage of the disease at diagnosis according to the topographic location of cancer, no differences were observed; the majority of patients were diagnosed at advanced stages (*p* = 0.08) (Table 3). HPV status was unknown for 94.1% of sampled patients; 3% were HPV (+) and 3% HPV (−). The recurrence rate was 13.9%. Local recurrence was found in 8.9% of cases, while 3.9% had metastasis; for two this occurred in another region of the head and neck and two had lung metastases (Table 2).

### 2.3. Association between Prior Use of Statins and Risk of Head and Neck Cancer

**Figure 1:**
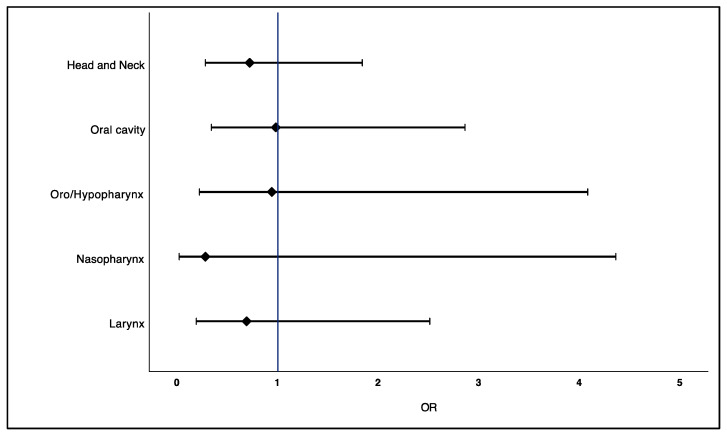
Adjusted OR for the association between statin exposure and head and neck cancer risk according to topographic location (Head and neck: OR = 0.72, 95% CI 0.28–1.84 (*p* = 0.49); Oral cavity: OR = 0.98, 95% CI 0.34–2.86 (*p* = 0.97); Oro/Hypopharynx: OR = 0.94, 95%CI 0.22–4.08 (*p* = 0.93); Nasopharynx: OR = 0.28, 95% CI 0.02–4.36 (*p* = 0.36); Larynx: OR = 0.69, 95% CI 0.19–2.51 (*p* = 0.57)).

A history of statin exposure was found in 28.2% of the total sample (*n* = 202). Most participants had lipophilic statin use (25.2%) and an average usage time of 11 years (Table 4). The prevalence of statin exposure was higher in controls (30.7%) than in patients with head and neck cancer (25.7%). Controls also had a slightly longer usage time of statin use. Doses higher than 40 mg/day were more frequent in cases (13.9%) than in controls (7.9%) (Table 4). After adjusting the multivariate logistic regression models controlled for education, monthly income, smoking status, alcohol consumption, physical activity, diet, and cardiovascular disease, prior use of statins was not associated with head and neck cancer risk (OR = 0.72; 95% CI 0.28–1.84) (Table 4). When exploring by topographic location of head and neck cancer, statin exposure before the diagnosis was not associated with cancers of the oral cavity, oro/hypopharynx, nasopharynx and larynx (Figure 1). A null effect was found for cancers of the oral cavity (OR = 0.98; 95% CI 0.34–2.86) and for cancers of the oro/hypopharynx (OR = 0.94; 95% CI 0.22–4.08) (Figure 1). There was also no association when analyzing by type of statin exposure, with neither lipophilic statins (OR = 0.71; 95% CI 0.27–1.86) nor hydrophilic statins (OR = 0.82; 95% CI 0.06–10.9) associated with a lower risk of head and neck cancer (Table 4). Likewise, there was no dose- and/or time-dependent association (Table 4).

### 2.4. Association between Prior Use of Statins and Recurrence of Head and Neck Cancer

Statins exposure was similar between patients with recurrence of head and neck cancer (28.6%) and patients without recurrence of the disease (25.3%) (Table 5). Similar trends were found when exploring by type of statin and by time of statin use. Patients without recurrence of head and neck cancer were more likely to use higher doses of statins than patients with recurrence of the disease (16.5% vs. 7.1%, respectively) (Table 5). The adjusted logistic regression models showed that prior use of statins was not associated with a lower recurrence rate of head and neck cancer (OR = 0.80; 95% CI 0.15–4.19). Lipophilic statin use was also not associated with a lower recurrence rate (OR = 0.91; 95% CI 0.17–4.95). The effect of hydrophilic statins could not be assessed, as no patients with recurrence of head and neck cancer were taking hydrophilic statins. There was also no dose- and/or time-dependent association between statin exposure and recurrence of head and neck cancer (Table 5).

## 3. Discussion

In this case–control study, prior use of statins was not associated with head and neck cancer risk (OR = 0.72; 95% CI 0.28–1.84). There was also no dose- and/or time-dependent relationship. When exploring by topographic location, prior use of statins was not associated with lower odds of cancers of the oral cavity, oro/hypopharynx, nasopharynx, and larynx. Similar trends were observed when exploring the relationship between statin use and head and neck cancer recurrence (OR = 0.80; 95% CI 0.15–4.19).

The effect of statins on cancer outcomes remains controversial. Emerging evidence from observational studies suggests that statins could play a potential role in cancer chemoprevention, reducing the risk of several site-specific cancers such as prostate [19], gastric [20], esophageal [6], hepatocellular [21] and colorectal [22], but not on ovarian and endometrial cancer [23,24]. However, meta-analyses of randomized clinical trials have contradicted these findings, suggesting that the protective effects of statins on cancer risk reported in observational studies are confounded [25,26]. The findings from the meta-analyses by Dale et al. [25], which included 26 randomized clinical trials, show that statins did not reduce the incidence of cancer (OR = 1.02; 95% CI 0.97–1.07) or cancer mortality (OR = 1.01; 95% CI 0.93–1.09). They also found that no type of cancer was affected by statin use and no subtype of statin affected the risk of cancer [25]. Another systematic review of randomized clinical trials conducted by the Cholesterol Treatment Trialists’ Collaboration reached the same conclusions, establishing that statin therapy does not have an effect on the incidence (RR = 1.00; 95% CI 0.96–1.05) or the mortality of cancer (RR = 1.00; 95% CI 0.93–1.08) [26]. These results are consistent with a later systematic review, which reported that, in patients with advanced cancer, the addition of statins to standard cancer therapy does not improve survival (HR = 0.94; 95% CI 0.85–1.04) or progression-free survival (HR = 0.97; 95% CI 0.87–1.07). Furthermore, there was no benefit observed when comparing hydrophilic statins alone vs. lipophilic statins alone [27].

Although there is a large amount of evidence regarding the effects of statins on cancer outcomes, there is limited evidence regarding their potential beneficial effects on head and neck cancer [16]. In contradiction with our findings, Kao et al. reported a significant inverse association between prior statin exposure and head and neck cancer risk (OR = 0.86; 95% CI 0.77–0.95) and a dose-dependent association for higher cumulative doses of statins compared to the non-use of statins (OR = 0.82; 95% CI 0.72–0.93) [17]. Although both studies considered similar prognostic factors in their analyses, the differences in estimation may be due to the lack of adjustment for dietary habits, and confounding due to the unavailability of an accurate measure of smoking and alcohol consumption history [17]. Another explanation could be related to the different population under study, which differs in genetic, environmental, and behavioral factors and which could influence the associations observed between statin exposure and head and neck cancer risk [28].

We also did not find an association between type of statin and head and neck cancer risk. Given that lipophilic statins can easily enter cells and interact with cell membranes while hydrophilic statins have greater hepatoselectivity and an impaired ability to penetrate biological membranes, it was presumed that they might provide different effects, as has been seen with cardiovascular outcomes [29]. Our results are consistent with a previous systematic review which showed no association between hydrophilic (RR = 1.00; 95% CI 0.82–1.17) and lipophilic statins (RR = 0.90; 95% CI 0.72–1.05) with the risk of prostate cancer [30] and with those reported by Dale et al. on overall cancer incidence (Hydrophilic: OR = 1.01, 95% CI 0.93–1.09; Lipophilic: OR = 1.01, 95% CI 0.97–1.11) [25].

Nevertheless, the results of a mendelian randomization study that assessed the causal effect of cholesterol-lowering on head and neck cancer risk found limited evidence for the role of cholesterol-lowering in oral cancer (OR = 1.49; 95% CI 0.75–2.96) and oropharyngeal cancer (OR = 0.90; 95% CI 0.43–1.85) [31]. The findings show an absence of a protective effect of genetically proxied inhibition of HMG-CoA reductase (statins) on head and neck cancer risk, further supporting the previous findings from randomized clinical trials. In line with this, Dickerman et al. [32] used the electronic health records of 733,804 UK adults followed for 10 years to emulate a target trial of statins and cancer. A pre-specified protocol was implemented to estimate the effect of statins on cancer incidence, including eligibility criteria, and verifications were made to ensure that the effect estimates for statins on cancer were comparable between the observational dataset and the target trial. Results from the intention-to-treat analysis showed a 10-year cancer-free survival difference between statin therapy vs. no statin therapy of −0.5% (95% CI −1.0–0.0%) (HR = 1.02; 95% CI 0.99–1.05). Per-protocol analysis showed the same results (−0.3%, 95% CI −1.5–0.5%; HR = 1.01, 95% CI 0.96–1.06), and cancer-free survival curves under each strategy were almost overlapping [32].

There are also a number of cohort studies that have reported that statin use is associated with improved overall survival and cancer-specific survival in patients with head and neck cancer [33,34]. However, the analyses were not adjusted for factors known to influence the prognosis of head and neck cancer such as tumor stage, HPV status as well as alcohol and smoking history [35,36]. In accordance with our results, Getz et al. [37] did not find an association between statin use and head and neck cancer recurrence (HR = 0.84 95% CI 0.69–1.02). When examining participants with HPV-positive and HPV-negative tumors separately, a protective relationship was observed for the HPV-positive patient’s rate of recurrence taking statins (HR = 0.49 95% CI 0.29–0.84) while a null association was observed for HPV-negative patients (HR = 1.03 95% CI 0.74–1.43) [37]. This stratified analysis further supports the finding that HPV status is a major predictor of prognosis in head and neck cancer outcomes, and that statins probably have a null effect; therefore, observed associations in previous studies may, in part, be confounded by HPV status. It has been reported that the two-year progression-free survival rates for HPV-positive and negative HNSCC range from 72–86% and from 50–75%, respectively [38]. In our study, we could not assess the effect of HPV in the recurrence rate of head and neck cancer as only 5.9% of patients had a reported status. A consensus to guide practitioners when to test for HPV has recently been reached with the guidelines from the College of American Pathologists published in 2018 [39]. Regardless of this, knowing and assessing for HPV status would have modified the effect size towards null, so there are no alterations in the qualitative interpretation of our findings.

Lipophilic statins were also not associated with the recurrence rate of head and neck cancer (OR = 0.91 95% CI 0.97–4.95). The results are in agreement with those reported by Ceacareanu et al., who found no beneficial effect of lipophilic statins on disease-free survival in patients with type 2 diabetes mellitus and solid tumors (HR = 0.91 95% CI 0.76–1.07) [40]. Conflicting results have been reported in preclinical studies. Hydrophilic rosuvastatin was found to be less effective in suppressing cancer cell growth than lipophilic atorvastatin. Pravastatin, another hydrophilic statin, has been found to not influence the growth of cancer cells [41]. The superiority of lipophilic statins at suppressing micro-metastatic outgrowth is attributed to their increased uptake into cancer cells [41]. Additionally, growth suppression by atorvastatin was found to be significantly potentiated by the inhibition of the PI3K-Akt pathway [41]. The prevalence of mutated PIK3CA and Akt have been estimated at 13% and 2% among head and neck cancer cell lines samples worldwide, respectively [42]. This may provide an explanation for the fact that different tumor cell lines exhibit differential relative sensitivities to lipophilic atorvastatin [43]. Moreover, statin concentrations used in in-vitro studies are difficult to correlate with human doses [41], which could account for the lack of association seen in observational studies.

Our findings have several limitations. The retrospective nature of the study makes it susceptible to selection bias, recall bias, and difficulties in adequately measuring exposure history. In an effort to minimize these sources of bias, we designed a structured survey that allowed us to measure those prognostic factors that influence the incidence of head and neck cancer, such as smoking, alcohol, and dietary habits [44]; and these were included in the logistic regression models. Regarding the temporality criterion, we considered statin exposure for at least one year before cancer diagnosis. Participants were asked for their medication prescription records to accurately register their history of statin exposure, in order to minimize recall bias. Although cases and controls were matched by age and sex, and the estimated ORs were adjusted for those risk factors that are known to influence the incidence of head and neck cancer, residual confounding cannot be ruled out. Even though controls were hospital-based, they come from the same source and geographic location as cases. These types of controls are frequently used in case–control studies as they share the same selection processes by which cases are identified, making this a more efficient design. Furthermore, the same data collection processes were used to minimize bias.

Another limitation is related to the sample size. We acknowledge that the estimated sample size resulted in a relatively small sample of participants when compared to other studies. However, an a priori sample size calculation was performed considering a significance level of 5% and a power of 80%, based on previous data, to estimate the association between statin exposure and head and neck cancer risk (detailed in the Methods section). Estimated sample size resulted in 196 participants, 98 cases and 98 controls, and we finally included 202 participants, 101 cases and 101 controls. Within our available resources, we designed and conducted the study trying to minimize potential sources of bias in order to ensure the internal validity and the reproducibility of the study, used a structured questionnaire for collecting the data, designed specifically for the study purpose, and used a sample of participants derived from the same part of the population. Analyses regarding the effects of statins among subsites of head and neck cancer and the recurrence rate should be interpreted with caution, since they were exploratory in nature.

As head and neck cancers are not a common outcome, randomized controls trials are less feasible, so we must partially rely on observational data. With a rise in research on repurposing drugs for the prevention and treatment of different cancers, the question that first arises is what type and amount of research needs to be presented to qualify evidence as strong for clinical practice implications and for decision-making. We believe that an adequate approach to address this would be the analysis of observational data by emulating a randomized clinical trial.

## 4. Materials and Methods

### 4.1. Design and Study Population

This was a hospital-based case–control study. The study population consisted of patients, older than 18 years, treated at the Dental Hospital of the University of Barcelona (HOUB) at the Faculty of Medicine and Health Sciences (Dentistry), Bellvitge campus, during 2018–2021. The research project was approved by the Clinical Research Ethics Committee (CEIC) of the Dental Hospital of the University of Barcelona in March 2018 (Approval code = 05-2018).

### 4.2. Definition of Cases and Controls

Cases were patients treated at the HOUB, in the Master of Dentistry in Oncology and Immunocompromised Patients of the University of Barcelona, during the period 2018–2021, diagnosed with head and neck cancer by histological confirmation (ICD C00-C14, C30-C32) and registration in the GESDEN^®^ database. We considered incident cases of head and neck cancer diagnosed since 2014. Inclusion criteria were that patients be older than 18 years, capable of filling in a questionnaire and that they gave their informed consent to participate in the study. Exclusion criteria ruled out patients who could not be explored or surveyed for medical reasons and patients with a previous diagnosis of another type of cancer.

Controls were patients treated at the HOUB, in the Master of Medicine, Surgery and Oral Implantology, or those who visited the Dentistry degree during the period 2014–2021, consecutively selected according to the eligibility criteria. Controls were matched to cases by age (±10 years) and sex in a 1:1 ratio. Inclusion criteria were that patients be older than 18 years, have no previous history of cancer, and be able to fill in a questionnaire, and that they gave their informed consent to participate in the study. Exclusion criteria ruled out patients who could not be explored or surveyed for medical reasons, patients with a previous history of head and neck cancer or another type of cancer, and patients with potentially malignant oral lesions.

### 4.3. Data Source, Identification of Cases and Controls and Recruitment

Cases and controls were identified through the electronic medical records of the GESDEN^®^ program, implemented in the HOUB since 2014, set as the index day. Cases were patients who usually were referred from the Functional Unit of Head and Neck at the Bellvitge’s Hospital and the Catalan Institute of Oncology, both in the L´Hospitalet of Llobregat location. Controls were individuals from the geographic location of L´Hospitalet of Llobregat, who attended the Master of Medicine, Surgery, and Oral Implantology or the Dentistry degree at the HOUB. Both cases and controls who met the eligibility criteria were consecutively contacted and invited to participate. All patients who were willing to participate in the study and gave their informed consent were included in the study, and a one-day visit was scheduled to the HOUB, where the study was conducted.

### 4.4. Sample Size

Given that at the time the study was designed, there were no prior published data regarding the association between statin use and head and neck cancer risk, a sample size calculation was performed based on the findings by Figueiredo et al. [45] on the association between metformin use and head and neck cancer risk. For an OR = 0.54 (95% CI 0.29–0.99), considering a 5% significance level, 80% of power and a 2-tailed test, the total sample size was estimated as 196 participants, 98 cases and 98 controls. Sample size calculation was performed with the G*Power 3.1 program.

### 4.5. Data Collection

Data collection was carried out through a structured survey designed for research purposes. Through the questionnaire, data on sociodemographic characteristics, history of tobacco, alcohol consumption, dietary habits, physical activity, and comorbidities were recorded. To avoid recall bias, patients were asked to provide their medical records on the diagnosis and treatment received (cases) and their regular medication prescriptions on the scheduled day to the HOUB. The type of medication, dose (mg/day) and time (years) of consumption were recorded. In patients with head and neck cancer, the topographic location of the cancer according to the 10th revision of the International Classification of Diseases (ICD C00-C14, C30-C32), date of diagnosis, TNM stage, HPV status, treatment received, and disease recurrence were recorded.

### 4.6. Exposure: Statins

Statin users were defined as those with a regular statin use for at least 1 year. Cases were considered statin users if they had a regular drug prescription for at least one year before cancer diagnosis. Those who started statin treatment at or after cancer diagnosis were considered non-statin users. Controls were considered statin users if they had a regular drug prescription for at least one year before the date of visiting the HOUB, identified through the GESDEN^®^ records. Those who started a regular consumption of statins less than one year from that date were considered non-statin-users. Type of statin (lipophilic and hydrophilic), regular dose (<10 mg/day, 20 mg/day, ≥40 mg/day) and time, in years, were considered to explore whether there was a dose- and/or time-dependent relationship. For cases, the duration in years, was calculated between the year of starting statin use and the year of cancer diagnosis, for controls, between the year of beginning statin use and the year of visiting the HOUB in the GESDEN^®^ registers.

### 4.7. Covariates

Cases and controls were matched for sex and age in three categories: 45–54 years, 55–64 years, and ≥65 years. Educational level was classified into four categories: without education, primary education, secondary education, and higher education. Monthly salary was classified in three categories according to the type of monthly income: low income (minimum monthly salary = 736 EUR/816 USD in 2018), middle income (2–4 times the minimum monthly salary), and high income (>4 times the minimum monthly salary). Smoking status was recorded in categorizing participants as non-smokers, ex-smokers, occasional smokers (≤1 cigarette/day), and current smokers. Furthermore, the number of cigarettes smoked per day in current and former smokers was recorded (<5 cigarettes/day, 5–10 cigarettes/day, 10–20 cigarettes/day, and >20 cigarettes/day). Alcohol consumption was measured in standard beverage units (1 SBU = 10 gm of ethanol). In men, moderate alcohol consumption was defined as a consumption equivalent to 2 SBUs or less per day (≤2 glasses of wine, 2 fifths of beer, or 1 glass of spirits). In women and those over 65 years, the equivalent of 1 SBU per day (≤1 glass of wine, 1 fifth of beer, or half a glass of spirits). Participants were categorized into alcohol-never-consumed, ex-consumers of alcoholic beverages, moderate alcohol consumption, and excessive alcohol consumption. Comorbidities were recorded as a self-reported diagnosis by a physician or health professional of type 2 diabetes mellitus, high blood pressure, and cardiovascular disease. Dietary habits were measured using a healthy-diet questionnaire adapted from the Spanish Society of Atherosclerosis, which has 14 questions with a yes/no answer and classifies the type of diet as unhealthy diet (≤4 points), regular diet (5–9 points) and healthy diet (10–14 points) [46]. Moderate physical activity, such as brisk walking, for 30 min or more each time in the last month was recorded, classifying participants into three categories: no practice of physical activity, 1–2 times per week, and ≥3 times per week.

### 4.8. Statistical Analysis

Data were collected through the Excel Corporation program (Microsoft, Redmond, WA, USA). Categorical variables were described by frequency and percentage, numerical variables according to their distribution, as mean and standard deviation or median and minimum-maximum. Bivariate associations between categorical variables were assessed with the chi-square test. Comparison between numerical and categorical variables was carried out according to the distribution of the numerical variable at each level of the categorical variable. For a normal distribution of the numerical variable, student´s t-tests or ANOVA was used, and in cases of a non-parametric distribution, the Mann–Whitney or Kruskal–Wallis tests were used.

Multivariate logistic regression models were adjusted to assess the association between prior use of statins and head and neck cancer risk. Models were adjusted for educational level, monthly income, smoking status, alcohol consumption, dietary habits, and physical activity. The variables age and sex were not included in the models, since they were balanced by design by matching cases and controls. Furthermore, multinomial logistic regression models were adjusted to explore the relationship between prior use of statins and the risk of head and neck cancer by topographic location (oral cavity, oro/hypopharynx, nasopharynx, and larynx), and logistic regression models to explore the association between statin exposure and recurrence of head and neck cancer. The reported measure of association was the Odds Ratio (OR) with 95% confidence intervals. A *p*-value < 0.05 was considered statistically significant. All analyses were performed with the program SPSS Statistics version 26 (IBM Corporation, Armonk, NY, USA).

## 5. Conclusions

Our findings do not support reports of a beneficial effect of prior statin exposure on head and neck cancer risk and the recurrence rate of head and neck cancer. There was also no effect by type of statin use and no time or dose-dependent association. Future studies relying on observational data should emulate randomized clinical trials before clinical implications for repurposing the use of statins on head and neck cancer prevention and treatment can be drawn.

## Figures and Tables

**Table 1:** Sociodemographic characteristics of cases and controls and factors associated with head and neck cancer.

Characteristics	Total Sample (*n* = 202) *n* (%)	Cases (*n* = 101) *n* (%)	Controls (*n* = 101) *n* (%)	Adjusted OR ^†^ (95% CI)	*p* Value
Sex					
Male	138 (68.3)	69 (68.3)	69 (68.3)		
Female	64 (31.7)	32 (31.7)	32 (31.7)		
Age (years)		66 ± 10.2 *	65.5 ± 9.8 *		0.73
44–54 years	30 (14.9)	15 (14.9)	15 (14.9)		
55–64 years	58 (28.7)	29 (28.7)	29 (28.7)		
≥65 years	114 (56.4)	57 (56.4)	57 (56.4)		
Education					
Without education	11 (5.6)	9 (9.4)	2 (2)	2.58 (0.35–18.7)	0.61
Primary education	101 (51.3)	52 (54.2)	49 (48.5)	0.62 (0.22–1.78)	0.39
Secondary education	43 (21.8)	23 (24)	20 (19.8)	1.57 (0.52–4.74)	0.98
Higher education	42 (21.3)	12 (12.5)	30 (29.7)	Reference	
Monthly income					
Low income	71 (39)	48 (59.3)	23 (22.8)	39.4 (4.08–381.3)	0.03
Middle income	93 (51.1)	32 (39.5)	61 (60.4)	8.86 (1.01–78.0)	0.04
High income	18 (9.9)	1 (1.2)	17 (16.8)	Reference	
Smoking status					
Current smoker	28 (13.9)	12 (11.9)	16 (15.8)	0.76 (0.23–2.56)	0.44
Occasional smoker (<1 cigarette/day)	4 (2)	1 (1)	3 (3)	1.98 (0.16–23.9)	0.58
Ex-smoker	91 (45)	59 (58.4)	32 (31.7)	2.36 (1.01–5.57)	0.03
Never smoked	79 (39.1)	29 (28.7)	50 (49.5)	Reference	
Cigarettes/day in smokers and ex-smokers					
>20 cigarettes/day	47 (54)	40 (69)	7 (24.1)	8.66 (1.38–54.2)	0.02
10–20 cigarettes/day	15 (17.2)	6 (10.3)	9 (31)	0.38 (0.04–3.26)	0.16
5–10 cigarettes/day	11 (12.6)	6 (10.3)	5 (17.2)	1.92 (0.22–16.4)	0.82
<5 cigarettes/day	14 (16.1)	6 (10.3)	8 (27.6)	Reference	
Alcohol consumption					
Excessive alcohol consumption	28 (13.9)	24 (23.8)	4 (4)	10.1 (2.10–49.3)	0.01
Moderate alcohol consumption	111 (55.2)	40 (39.6)	71 (71)	1.04 (0.36–2.98)	0.82
Ex-consumer of alcoholic beverages	16 (8)	13 (12.9)	3 (3)	3.62 (0.69–18.9)	0.28
Alcohol never consumed	46 (22.9)	24 (23.8)	22 (22)	Reference	
Comorbidities					
Diabetes Mellitus 2	36 (17.8)	12 (11.9)	24 (23.8)	0.69 (0.26–1.82)	0.62
High blood pressure	88 (43.6)	39 (38.6)	49 (48.5)	0.90 (0.19–4.36)	0.87
Cardiovascular disease	32 (15.8)	21 (20.8)	11 (10.9)	1.66 (0.23–12.2)	0.61
Physical activity					
≥3 times per week	64 (34.2)	26 (30.2)	38 (37.6)	0.42 (0.16–1.11)	0.16
1–2 times per week	55 (29.4)	19 (22.1)	36 (35.6)	0.46 (0.18–1.21)	0.14
No practice of physical activity	68 (36.4)	41 (47.7)	27 (26.7)	Reference	
Diet					
Healthy diet	49 (27.2)	10 (12.7)	39 (38.6)	0.29 (0.10–0.84)	0.005
Regular diet	72 (40)	32 (40.5)	40 (39.6)	0.77 (0.32–1.86)	0.38
Unhealthy diet	59 (32.8)	37 (46.8)	22 (21.8)	Reference	

* Mean ± standard deviation, ^†^ Model. Response variable: cases. Covariates: education, monthly salary, smoking status, cigarettes/day, alcohol, physical activity, and diet.

**Table 2:** Cancer history of patients with head and neck cancer.

Oncological History	Cases (*n* = 101)
Topographic location	
Oral cavity	49.5%
Oro/Hypopharynx	18.8%
Nasopharynx	5%
Larynx	26.7%
TNM staging	
I	9.9%
II	10.9%
III	22.8%
IVA	33.7%
IVB	5.9%
HPV status	
HPV (+)	3%
HPV (−)	3%
Unknown	94.1%
Cancer treatment	
Surgery	15.8%
Surgery + radiotherapy	20.8%
Induction chemotherapy + surgery + radiotherapy	17.8%
Induction chemotherapy + radiotherapy	37.6%
Radiotherapy + biological therapy (cetuximab)	5.9%
Other (brachytherapy)	2.0%
Recurrence rate	13.9%
Local recurrence	8.9%
Metastasis	3.9%

**Table 3:** TNM staging of head and neck cancer patients by topographic location.

	Topographic Location			
TNM Staging	Oral Cavity (*n* = 50) *n* (%)	Oro/Hypopharynx (*n* = 19) *n* (%)	Nasopharynx (*n* = 5) *n* (%)	Larynx (*n* = 27) *n* (%)
I or II	14 (33.3)	5 (33.3)	0 (0)	2 (9.1)
III or IV	28 (66.7)	10 (66.7)	5 (100)	20 (90.9)

**Table 4:** Prevalence of statin exposure among cases and controls and adjusted logistic regression models (OR) for the association between statin exposure and head and neck cancer risk.

Statin Exposure	Total Sample (*n* = 202) *n* (%)	Cases (*n* = 101) *n* (%)	Controls (*n* = 101) *n* (%)	Crude OR (95% CI) *n* (%)	Adjusted OR ^†^ (95% CI)	*p* Value
Overall						
Non-use of statins	145 (71.8)	75 (74.3)	70 (69.3)	Reference	Reference	
Regular use of statins	57 (28.2)	26 (25.7)	31 (30.7)	0.78 (0.42–1.45)	0.72 (0.28–1.84)	0.49
Type of statins						
Lipophilic	51 (25.2)	22 (21.8)	29 (28.7)	0.71 (0.37–1.34)	0.71 (0.27–1.86)	0.49
Hydrophilic	6 (3)	4 (4)	2 (2)	1.86 (0.33–10.5)	0.82 (0.06–10.9)	0.88
Daily dose						
≤10 mg/day	12 (5.9)	3 (3)	9 (8.9)	0.31 (0.09–1.19)	0.39 (0.06–2.67)	0.34
20 mg/day	23 (11.4)	9 (8.9)	14 (13.9)	0.60 (0.24–1.47)	0.62 (0.16–2.37)	0.48
≥40 mg/day	22 (10.9)	14 (13.9)	8 (7.9)	1.63 (0.65–4.13)	1.13 (0.30–4.22)	0.85
Time (years)	11.4 ± 8.3 *	10 ± 9.1 *	12.7 ± 7.5 *	0.96 (0.89–1.03)	0.85 (0.69–1.04)	0.11

* Mean ± standard deviation. ^†^ Adjusted for education, monthly income, smoking status, alcohol consumption, diet, physical activity, and cardiovascular disease.

**Table 5:** Association between statin exposure and recurrence of head and neck cancer.

Statin Exposure	Recurrence (*n* = 14) *n* (%)	No Recurrence (*n* = 79) *n* (%)	Crude OR (95% CI)	Adjusted OR ^†^ (95% CI)	*p* Value
Overall					
Non-use of statins	10 (71.4)	50 (74.7)	Reference	Reference	
Regular use of statins	4 (28.6)	20 (25.3)	1.18 (0.33–4.18)	0.80 (0.15–4.19)	0.79
Type of statin					
Lipophilic	4 (28.6)	17 (21.5)	1.39 (0.39–4.98)	0.91 (0.17–4.95)	0.91
Hydrophilic	0 (0)	3 (3.8)	Not estimable	Not estimable	
Daily dose					
≤10 mg/day	1 (7.1)	1 (1.3)	5.90 (0.34–102.1)	4.43 (0.12–168.2)	0.42
20 mg/day	2 (14.3)	6 (7.6)	1.96 (0.35–11.1)	1.98 (0.14–28.5)	0.61
≥40 mg/day	1 (7.1)	13 (16.5)	0.45 (0.05–3.86)	0.32 (0.03–3.48)	0.32
Time (years)	10 ± 8.4	10.2 ± 9.6	0.99 (0.85–1.17)	0.99 (0.82–1.20)	0.95

^†^ Adjusted for education, monthly income, smoking status, alcohol consumption, diet, and stage at diagnosis.

## Data Availability

Data is contained within the article.
